# Supplementary material for: The Elevated Inflammatory Status of Neutrophils Is Related to In-Hospital Complications in Patients with Acute Coronary Syndrome and Has Important Prognosis Value for Diabetic Patients
Source: Int J Mol Sci. 2024 May 8;25(10):5107. doi: 10.3390/ijms25105107 (PMC11121518; doi:10.3390/ijms25105107)
Supplement: Supplementary file 1 [file ijms-25-05107-s001.zip › ijms-2975066-supplementary.pdf]

Supplementary Table S1:

Table S1. The sequences of oligonucleotide primers used for evaluation of gene expression.

**Gene (Human)**	**Sequences of Oligonucleotide Primers**
CCL3	FW: AGTTCTCTGCATCACTTGCTG<br>RV: CCGCTTCGCTTGGTTAGGAA
IL-1β	FW: AGAGTGGAGCCTGGTCTTACA<br>RV: CCTTTGCTGACAATAAGCACTGG
IL-18	FW: GATAGCCAGCCTAGAGGTATGG<br>RV: CCTTGATGTTATCAGGAGGATTCA
S100A9	FW: TCCTCGGCTTTGACAGAGTG<br>RV: GCCCCAGCTTCACAGAGTAT
IL-6	FW: CTGCAGAATTCCAGGACCACA<br>RV: TCCGGTGGTGTAAAGAGGAC
ICAM-1	FW: GGTTCTTCTGAGCGGCGTCG<br>RV: CCAGCCGAGGACCATACAGC
p22phox	FW: CCCAGTGGTACTTTGGTGCC<br>RV: GCGGTCATGTACTTCTGTCCC
Nox2	FW: ACCGGGTTTATGATATTCCACCT<br>RV: GATTTCGACAGACTGGCAAGA
MMP-9	FW: AGACCTGGGCAGATTCCAAAC<br>RV: CGGCAAGTCTTCCGAGTAGT
TLR4	FW: CACACCGTCATCAGCATTGA<br>RV: CTCTGGCGTAGAGCTATCACT

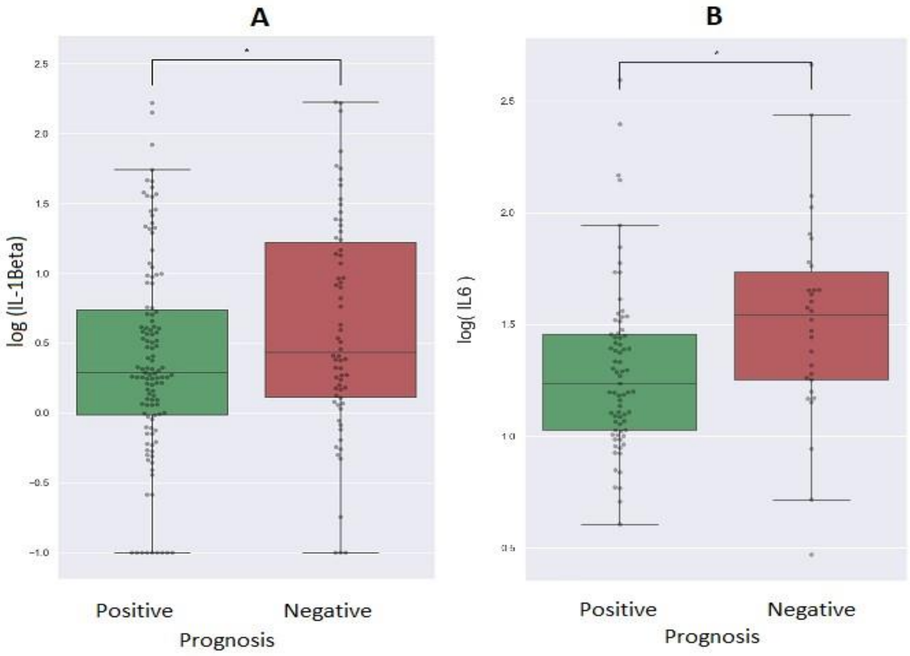

Supplementary Figure S1. *Box plots showing the comparison of Interleukine Beta 1 (IL1-Beta)* ***(A)*** *and Interleukine 6 (IL-6)* ***(B) l****evels between patients with favorable vs unfavorable prognosis (Student t test, *p<0.05).*

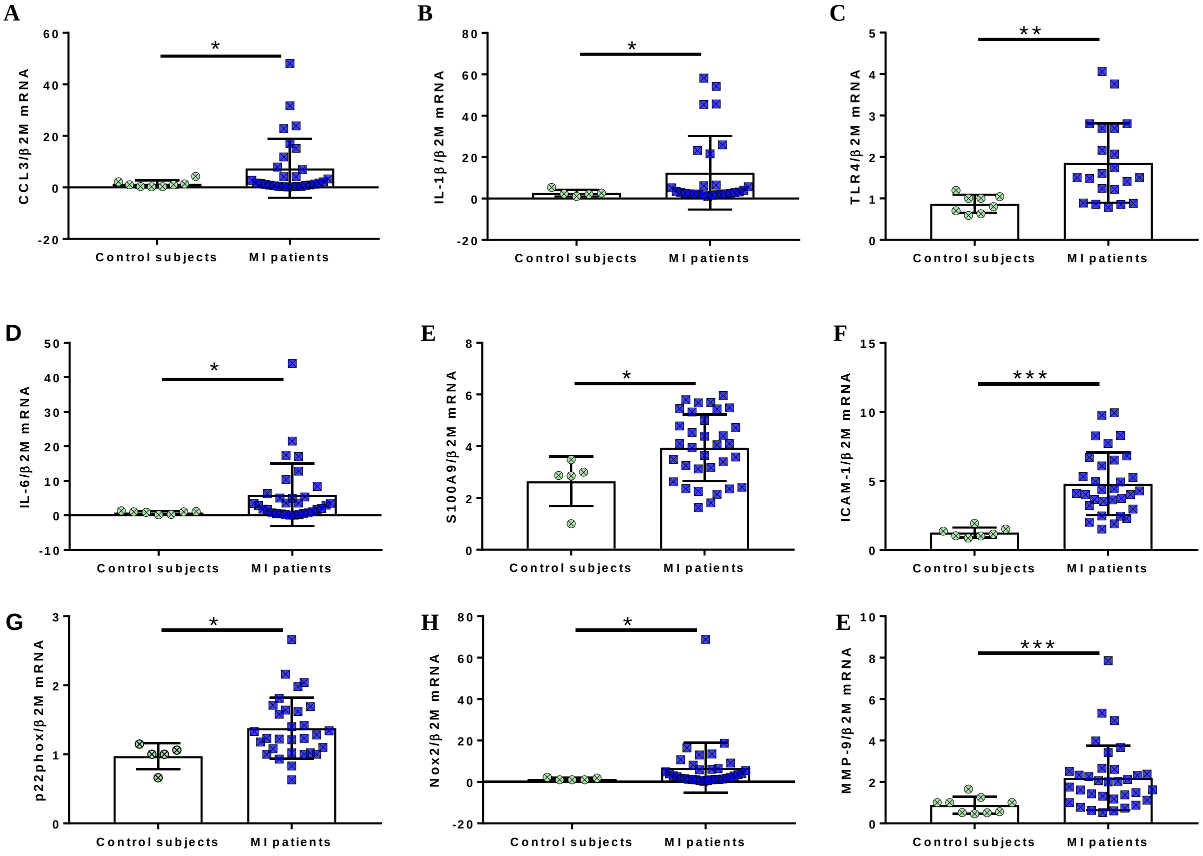

Supplementary Figure S2. ***Gene expression of inflammatory molecules in MI patients versus health controls.*** *Neutrophils from MI patients exhibit increased expression of all investigated genes. (**A-E**) The expression of genes associated with an inflammatory phenotype of neutrophils CCL3, IL-1β, IL-18, IL-6, S100A9, ICAM-1, as well as of p22phox, Nox-2, and MMP-9 were investigated in neutrophils from control healthy subjects or patients with MI (M_NP). *p < 0.05, **p < 0.01, ***p < 0.001 (control vs. MI).*

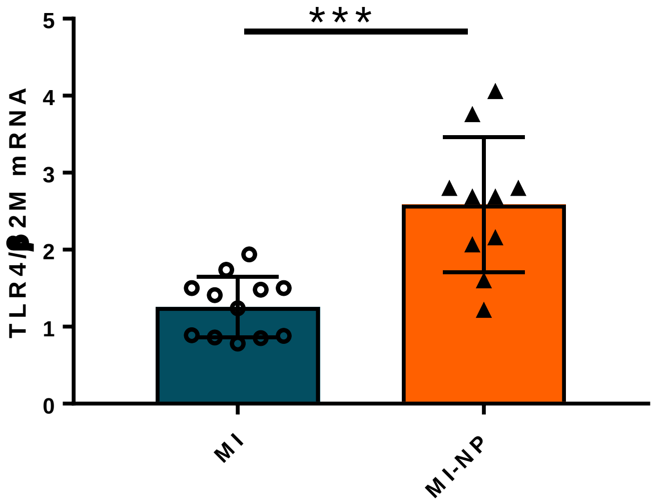

Supplementary Figure S3. ***Gene expression of TLR4 in MI patients with or without negative prognosis.*** *Neutrophils from ACS patients with negative prognostic exhibit increased expression of TLR4 in neutrophils isolated within first 24h after MI onset., ***p < 0.001, (MI_NP vs. MI).*
